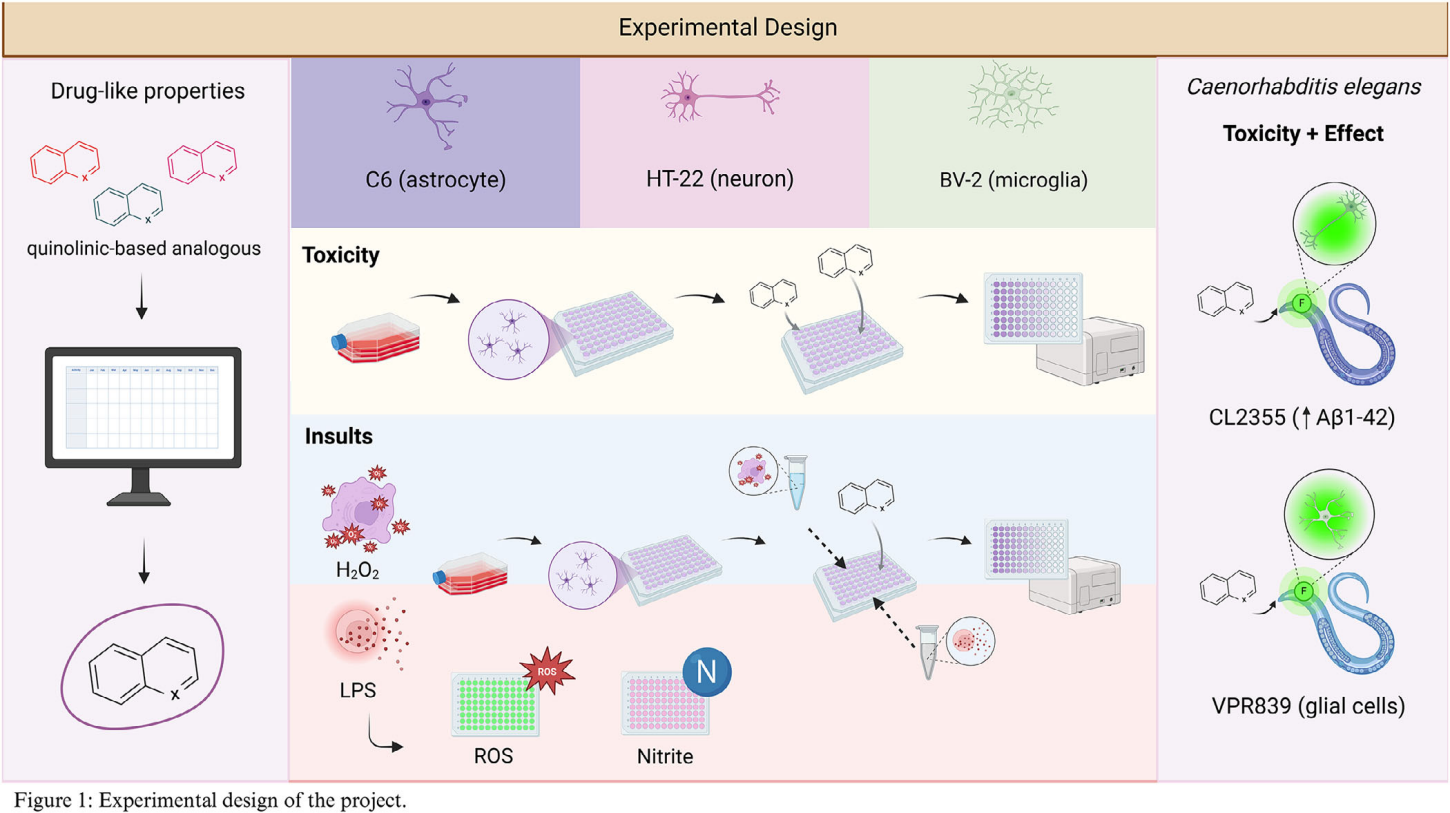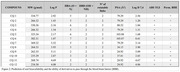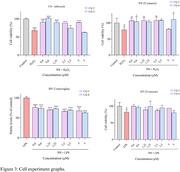# Heterocyclic Quinoline Derivatives as Innovative Neuroprotective Agents for Alzheimer's Disease

**DOI:** 10.1002/alz70859_104682

**Published:** 2025-12-25

**Authors:** Maria Paula Faccin Huth, Angelica Rocha Joaquim, Marcela Silva Lopes, Karine Rigon Zimmer, Saulo Fernandes de Andrade, Aline R. Zimmer

**Affiliations:** ^1^ Federal University of Rio Grande do Sul, Porto Alegre, RS Brazil; ^2^ Federal University of Santa Maria, Santa Maria, RS Brazil; ^3^ Universidade Federal de Ciencias da Saude de Porto Alegre, Porto Alegre, Rio Grande do Sul Brazil; ^4^ Federal University of Rio Grande do Sul, Porto Alegre, Rio Grande do Sul Brazil; ^5^ McGill, Montreal, QC Canada

## Abstract

Heterocyclic Quinoline Derivatives as Novel Neuroprotective Agents for Alzheimer's Disease

**Background:**

Alzheimer's disease (AD) is a complex multifactorial neurodegenerative disease and the leading cause of dementia. Different pathological processes contribute to the disease's progress, and a multitarget approach is considered an attractive strategy for drug design and discovery in AD. Quinolines are a class of heterocyclic compounds with diverse biological activities, especially chelating properties, antioxidants, and reducing brain Aβ levels. Quinolinic scaffolds have been explored as potential applications in the treatment of AD. In this study, quinolinic‐based analogous has been designed targeting anti‐inflammatory and antioxidant activity to be used at the early stages of Alzheimer's disease and reduce the disease's onset and progression.

**Method:**

We designed a series of quinolinic‐based compounds and evaluated the drug‐like properties and the blood‐brain barrier (BBB) permeability using *in silico* virtual platforms. The cytotoxicity and neuroprotective effects of the most promising derivatives were tested in *in vitro* assays using three different brain cell lines (BV‐2 microglial, C6 astroglial, and HT‐22 neuronal cells) against inflammatory (LPS) and oxidative (H2O2) insults. The effects on the production of nitrite and reactive oxygen species were determined. We also tested the toxicity and effects of the compounds on the in vivo *Caenorhabditis elegans* transgenic model *(*CL2355, neurons expressing Aβ1‐42, and VPR839 strains).

**Result:**

All molecules have shown high potential for oral absorption, and 80% are promising to cross BBB. The heterocyclic derivatives that exhibited IC50 values higher than 50 µM in all tested brain cells were classified as low toxicity and chosen for neuroprotection assays. Two quinolinic derivatives successfully protected the glial and neuronal cells of oxidative insults and showed a tendency towards neuroinflammatory protection in neuronal cells, in concentrations lower than 5 µM. These compounds reduced nitric oxide levels by 30%. The studies with the *C. elgans* fluorescent strains are in progress.

**Conclusion:**

The designed heterocyclic‐based derivative showed physicochemical characteristics compatible with oral use and passage through BBB and relevant antioxidant and anti‐inflammatory effects. These findings suggest these compounds are promising multitarget agents for preventing AD, offering opportunities for future drug design in this field.